# Clinical features and treatment outcomes of Castleman disease in children: a retrospective cohort in China

**DOI:** 10.1007/s00431-023-05235-2

**Published:** 2023-10-02

**Authors:** Shiwen Hu, Zifeng Li, Hongsheng Wang, Lian Chen, Yangyang Ma, Xiaohua Zhu, Jun Li, Rui Dong, Wei Yao, Chenbin Dong, Huifeng Zhang, Kai Li, Kuiran Dong, Xiaowen Zhai

**Affiliations:** 1 Department of Hematology and Oncology, Children’s Hospital of Fudan University, National Children’s Medical Center, Shanghai, China; 2 Department of Pathology, Children’s Hospital of Fudan University, National Children’s Medical Center, Shanghai, China; 3 Department of Pediatric Surgery, Children’s Hospital of Fudan University, National Children’s Medical Center, Shanghai, China; 4 Department of Cardiothoracic Surgery, Children’s Hospital of Fudan University, National Children’s Medical Center, Shanghai, China

**Keywords:** Castleman disease, Pediatrics, Unicentric, Multicentric

## Abstract

Castleman disease (CD) is a rare lymphoproliferative disorder of undetermined etiology. Unicentric CD (UCD) and multicentric CD (MCD) are two phenotypes of CD diagnosed by the histopathology of lymph nodes. We attempted to describe a pediatric CD cohort to optimize the management of this disease. We reviewed the medical records of pediatric patients diagnosed with CD between April, 2004, and October, 2022, at the Children’s Hospital of Fudan University. Prognosis information was collected in January, 2023, by telephone inquiry. Twenty-two patients with UCD and 2 patients with MCD were identified, all with hyaline vascular (HV) type. The median ages at diagnosis were 10.75 years (IQR 8, 12.81) for UCD and 14.42 years (IQR 13.42, 15.42) for MCD. The most common lesion location of UCD was the neck (9/22, 40.91%) and abdomen (9/22, 40.91%). Systematic symptoms occurred on 10/22 (45.45%) patients with UCD and 1/2 (50%) patients with MCD, and abnormal laboratory indexes were detected in both. Resection and biopsy were performed on all patients. One out of two patients with MCD also received rituximab for upfront therapy. After a median of 4 years (IQR 1.5, 6) of follow-up time, the overall survival was 100% and the complete remission rate in UCD was 63%. There was no relapse or progression.

*Conclusions*: Our series demonstrated that HV-UCD was the most common type in children. Resection and biopsy were used for both deterministic diagnoses and treatments. Despite the high possibility to develop systematic inflammation, children with CD showed promising outcomes.

**Table:** 

**What is Known:** *• Castleman disease is a rare lymphoproliferative disorder with limited cohort studies, especially in pediatrics.* *• The ubiquity of delayed confirmations and misdiagnoses points to a lack of knowledge about etiology and characteristics, which is a prerequisite for novel therapeutics.*	
**What is New:** *• We retrospectively reviewed and analyzed the clinical and pathological symptoms, laboratory and imaging features, and treatment outcomes of a Chinese pediatric cohort with Castleman disease.* *• Our work may improve the recognition and optimize the management of this rare disease in children.*

**What is Known:**

*• Castleman disease is a rare lymphoproliferative disorder with limited cohort studies, especially in pediatrics.*

*• The ubiquity of delayed confirmations and misdiagnoses points to a lack of knowledge about etiology and characteristics, which is a prerequisite for novel therapeutics.*

**What is New:**

*• We retrospectively reviewed and analyzed the clinical and pathological symptoms, laboratory and imaging features, and treatment outcomes of a Chinese pediatric cohort with Castleman disease.*

*• Our work may improve the recognition and optimize the management of this rare disease in children.*

## Introduction

Castleman disease (CD) is a lymphoproliferative disease of unidentified etiology, first reported in 1954 and named in 1956 by Castleman et al. [1]. CD has a low incidence of approximately 6500 to 7700 cases per year in the USA [2]; however, definitive reports of childhood morbidity are unavailable. CD can be classified as unicentric CD (UCD) or multicentric CD (MCD) according to its involvement and clinical course [3]. Based on the primary driver of the disease, MCD is further classified as idiopathic MCD (iMCD); human herpes virus 8 (HHV-8)-associated MCD (HHV-8-MCD); and polyneuropathy, organomegaly, endocrinopathy, monoclonal plasma cell disorder, skin changes (POEMS) syndrome-associated MCD (POEMS-MCD) [4]. Confirmation of the diagnosis relies on histopathological findings. The hyaline vascular (HV) type is characterized by capsular fibrosis, increased numbers of lymphoid follicles, and regressed germinal centers (GCs) penetrated by pathological hyaline blood vessels [2]. Additionally, hyperplastic mantle zones present a typical “onion skin pattern,” comprising concentric rings of small lymphocytes [2]. The plasma cell (PC) type is characterized by the enrichment of sheetlike PCs in the interfollicular zone, with hyperplastic GCs [3]. Features of both types are present in the mixed type.

The most frequent type of CD in children is HV-UCD, which commonly originates in the neck [5]. The predominant sign is an enlarged, painless lymph node. By contrast, MCD is more aggressive and accompanied by frequent systemic symptoms and laboratory abnormalities. UCD can usually be cured by surgical resection and has a good survival rate, whereas MCD has a worse prognosis because of the risk of relapse or progression. Both misdiagnosis and delayed diagnosis are common in children because of their unclear complaints and inadequate awareness among parents and pediatricians.

We conducted this retrospective study to analyze the clinical and pathological symptoms, laboratory and imaging features, and treatment outcomes of children with CD in an effort to improve the recognition and management of this rare disease.

## Methods

### Patients and materials

This study involved children diagnosed with CD based on the pathological evidence at the Children’s Hospital of Fudan University from April, 2004, to October, 2022. All the diagnoses were reviewed and confirmed by clinical experts, pathologists, and radiologists. The diagnostic delay was defined as the time from the first symptom or identification of a mass to diagnosis. The patients’ demographic information, clinical presentation, pathological findings, and first-line therapy were retrospectively collected from the medical records. Laboratory data and imaging observations prior to surgical resection were chosen for analysis; if several examinations were performed, the results of the first visit were selected.

### Follow-up

We were able to access prognostic information for 16 of 22 patients with UCD and 1 of 2 patients with MCD by telephone follow-up in January, 2023. Overall survival (OS) was defined as the time from diagnosis to relapse, death, or the last follow-up.

### Statistical analysis

The patients were stratified according to the disease subtype or whether they were asymptomatic. Categorical variables are presented as count and percentage, and continuous variables are presented as median (interquartile range [IQR]) or mean (standard deviation). Wilcoxon’s rank sum test or Student’s t-test was used to calculate the *P*-value, and *P* < 0.05 indicated statistical significance. Statistical analyses were performed using Stata/MP version 17.0 (StataCorp, College Station, TX, USA).

## Results

We identified 24 patients with UCD (*n* = 22) and MCD (*n* = 2) according to biopsy-proven histopathological features. The patients’ characteristics are shown in Tables 1 and 2.

**Table 1:** Characteristics, treatments, and outcomes, by disease subtype

-	UCD (*n* = 22) Median (IQR) or *n* (%)	MCD (*n* = 2) Median (IQR) or *n* (%)
Sex ratio (female/male)	11/11	0/2
Age at diagnosis (year)	10.75 (8, 12.81) (range: 2.08–16.08)	14.42 (13.42, 15.42) (range: 13.42–15.42)
Diagnosis delay (month)	5.25 (1, 15) (range: 0.17–52)	12.08 (0.17, 24) (range: 0.17–24)
Follow-up time (year)	4.08 (1.63, 6.08)	1.5
Primary lymph node location		
Neck/submaxilla	9 (40.91%)	1 (50%)
Axilla	1 (4.55%)	1 (50%)
Mediastinum	2 (9.09%)	0 (0%)
Abdomen	9 (40.91%)	0 (0%)
Groin	1 (4.55%)	0 (0%)
Pathological subtype		
Hyaline vascular	22 (100%)	2 (100%)
Plasma cell	0 (0%)	0 (0%)
Mixed	0 (0%)	0 (0%)
Clinical characteristics		
Fever	0 (0%)	1 (50%)
Anemia	10 (45.45%)	1 (50%)
Growth retardation	1 (4.55%)	0 (0%)
Fatigue	1 (4.55%)	0 (0%)
Anorexia	1 (4.55%)	0 (0%)
Abdominal pain or discomfort	3 (13.64%)	0 (0%)
Hepatomegaly	4/20 (20%)	1/2 (50%)
Splenomegaly	3/20 (15%)	0/2 (0%)
Laboratory characteristics		
Decreased albumin	7/21 (33.33%)	1/2 (50%)
Elevated globulin	12/21 (57.14%)	1/2 (50%)
Elevated C-reactive protein	5/15 (33.33%)	1/2 (50%)
Hemoglobin level, g/L	116 (97.55,132)	133 (107,159)
Decreased MCV	8/18 (44.44%)	0/2 (0%)
Platelet count, × 10^9^/L	307 (265.25,447)	289 (265,313)
HIV positivity	0/19 (0%)	0/2 (0%)
EBV IgG positivity	6/6 (100%)	2/2 (100%)
CMV IgG positivity	4/6 (66.67%)	2/2 (100%)
Treatment		
Surgery	22 (100%)	2 (100%)
Chemotherapy/radiotherapy	0 (0%)	0 (0%)
Rituximab	0 (0%)	1 (50%)
Outcomes		
Complete remission	10/16 (62.5%)	0 (0%)
Stable lymphadenopathy	6/16 (31.25%)	1/1 (100%)
Relapse/progression	0/16 (0%)	0 (0%)

*UCD *unicentric Castleman disease, *MCD *multicentric Castleman disease, *IQR *interquartile range, *MCV *mean corpuscular volume, *HIV *human immunodeficiency virus, *EBV *Epstein-Barr virus, *CMV *Cytomegalovirus

**Table 2:** Characteristics, treatments, and outcomes, by patients

Patient/sex	Disease Subtype	Age at diagnosis (year)	Diagnosis delay (month)	Follow-up time (year)	Primary lymph node location	Initial symptom	Path. subtype	Hb level (g/L)	MCV (fl)	Platelet count (× 10^9^/L)	CRP level (mg/L)	Albumin/ Globulin level, g/L	Diagnostic investigation	Upfront treatment	Outcome
P1/M	UCD	13	12	Loss	Neck	Asymptomatic	HV	133	-	178	-	-	-	Resection	-
P2/M	UCD	8	36	Loss	Neck	Asymptomatic	HV	136	-	269	-	50/30.6	Ultrasound, CT, FNAC, LN biopsy	Resection	-
P3/F	UCD	9	0.7	Loss	Abdomen	Abdominal pain	HV	115	-	254	-	44.9/32.3	Ultrasound, CT, LN biopsy	Resection	-
P4/F	UCD	8	4	Loss	Abdomen	Pallor	HV	106	60	608	-	33.7/51.9	Ultrasound, CT, MRI, LN biopsy	Resection	-
P5/F	UCD	10	4.5	Loss	Abdomen	Anemia	HV	104	-	282	-	33.1/42.4	Ultrasound, CT, MRI, LN biopsy	Resection	-
P6/M	UCD	13	12	10.42	Abdomen	Anemia, anorexia, growth retardation	HV	90	81	698	-	29.8/43.8	Ultrasound, CT, LN biopsy	Resection	Complete remission
P7/M	UCD	9	36	9.17	Abdomen	Pallor	HV	83	60	435	-	34.4/48.9	Ultrasound, CT, MRI, LN biopsy	Resection	Complete remission
P8/F	UCD	11	12	Loss	Mediastinum	Pallor, fatigue, frequent colds, recurrent nosebleeds	HV	81.2	69	641	> 160	27.7/84.9	Ultrasound, CT, MRI, LN biopsy	Resection	-
P9/F	UCD	9	0.33	7.08	Abdomen	Asymptomatic	HV	93.2	71	281	20	39.7/36.2	Ultrasound, MRI, LN biopsy	Resection	Complete remission
P10/F	UCD	11	2	6.17	Neck	Asymptomatic	HV	132	82	366	< 8	49.6/24.6	-	Resection	Stable lymphadenopathy, prone to get cold and fever
P11/M	UCD	16	52	5.83	Neck	Asymptomatic	HV	106	91	242	< 8	45.1/24.2	Ultrasound, CT, LN biopsy	Resection	Complete remission
P12/F	UCD	2	6	5.25	Groin	Asymptomatic	HV	130	76.1	272	< 8	45/23.4	Ultrasound, LN biopsy	Resection	Complete remission
P13/F	UCD	13	4	4.25	Neck	Asymptomatic	HV	133	84.5	226	< 8	40.6/33	Ultrasound, CT, LN biopsy	Resection	Complete remission
P14/M	UCD	3	24	4.17	Neck	Asymptomatic	HV	116	82.4	370	< 8	46.2/27.6	Ultrasound, FNAC, LN biopsy	Resection	Stable lymphadenopathy
P15/M	UCD	6	10	4	Neck	Asymptomatic	HV	130	79.6	442	< 8	39.8/25.1	Ultrasound, CT, LN biopsy	Resection	Complete remission
P16/F	UCD	13	1	3.33	Neck	Asymptomatic	HV	132	85.5	329	< 8	42.7/29.3	Ultrasound, CT, LN biopsy	Resection	Complete remission
P17/M	UCD	11	6	3	Submaxilla	Asymptomatic	HV	129	81.9	404	< 8	44.9/32.2	Ultrasound, CT, FNAC, LN biopsy	Resection	Stable lymphadenopathy
P18/M	UCD	12	36	2	Abdomen	Abdominal discomfort	HV	147	86.5	283	10	42.7/25.6	Ultrasound, CT, LN biopsy	Resection	Complete remission
P19/F	UCD	8	1	1.5	Abdomen	Chest pain	HV	78	60.6	462	117	31.1/31.6	Ultrasound, CT, LN biopsy	Resection	Complete remission
P20/M	UCD	2	1	1.25	Axilla	Asymptomatic	HV	116	76.5	252	< 8	46.97/20.63	Ultrasound, CT, LN biopsy	Resection	Stable lymphadenopathy
P21/M	UCD	12	0.5	0.42	Mediastinum	Asymptomatic	HV	131	77.3	285	< 8	45.13/ 27.57	Ultrasound, CT, MRI, LN biopsy	Resection	Stable lymphadenopathy, fatigue
P22/F	UCD	12	0.17	0.25	Abdomen	Abdominal pain, vomiting	HV	99	67.2	507	76	39.04/ 48.46	Ultrasound, CT, LN biopsy	Resection	Stable lymphadenopathy
P23/M	MCD	13	24	Loss	Neck	Asymptomatic	HV	159	87.3	265	< 8	47.1/27.8	Ultrasound, PET/CT, LN biopsy	-	-
P24/M	MCD	15	0.17	1.5	Axilla	Fever, anemia	HV	107	77.9	313	245.87	34.21/ 32.99	Ultrasound, CT, PET/CT, LN biopsy	Rituximab	Stable lymphadenopathy

*M *male, *F *female, *UCD *unicentric Castleman disease, *MCD *multicentric Castleman disease, *HV *hyaline vascular, *Hb *hemoglobin, *MCV *mean corpuscular volume, *CRP *C-reactive protein, *FNAC *fine-needle aspiration cytology, *LN *lymph node

### UCD

The sex ratio was 1:1, the median age at diagnosis was 10.75 years (IQR: 8, 12.81), and the median diagnostic delay was 5.25 months (IQR: 1, 15).

**Fig. 1:**
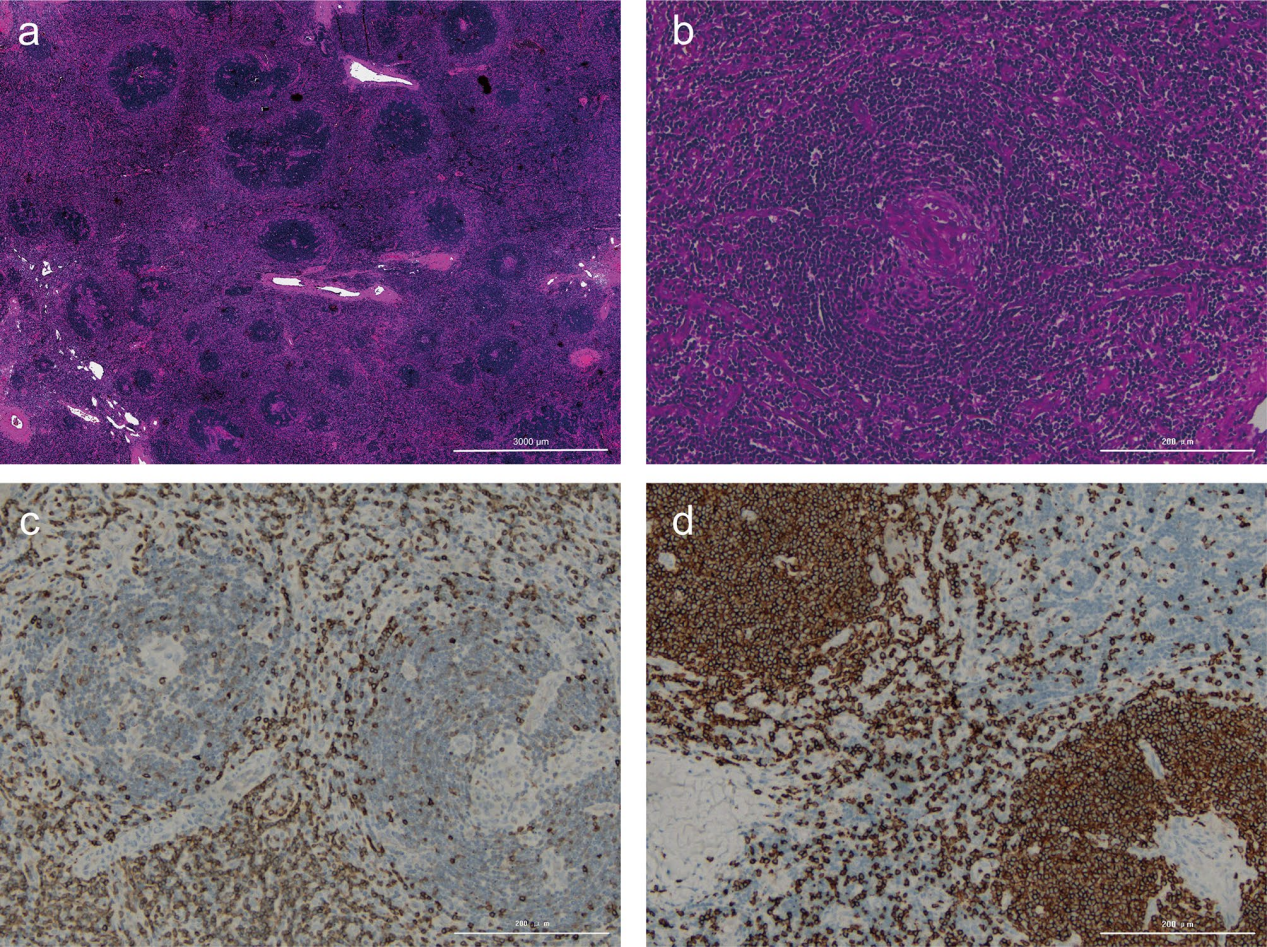
Representative histopathological findings of an involved lymph node in Castleman disease with hyaline vascular type. **a** Typical manifestations of hyperplastic follicles with regressive germinal centers and concentric proliferating lymphocytes; **b** a typical lymphatic follicle; **c** expression patterns of CD5; **d** expression patterns of CD20

All 22 patients underwent excisional biopsy and the results of the immunohistochemical (IHC) examination of 20 were available. All cases were histologically classified as the HV subtype. Bcl-2 and biomarkers of T lymphocytes, including CD3 and CD5, were detected in interfollicular areas, whereas Bcl-6 and biomarkers of B lymphocytes, including CD20 and CD79a, were detected within follicles. Representative histopathological findings are shown in Fig. 1.

The most common primary lymph nodes were located in the neck (9/22, 41%) and abdomen (9/22, 41%), followed by the mediastinum (2/22, 9%), axilla (1/22, 5%), and groin (1/22, 5%). Twelve (55%) cases were unintentionally palpated and 10 (45%) were incidentally discovered during imaging examinations for various reasons. At diagnosis, systemic symptoms were present in 10/22 (45%) patients, including anemia (10/22, 45%), growth retardation (1/22, 5%), fatigue (1/22, 5%), and anorexia (1/22, 5%). Other complaints were abdominal pain or discomfort (3/22), vomiting (1/22), chest pain (1/22), frequent colds (1/22), and recurrent nosebleeds (1/22).

Among the anemic children, 7/10 (70%) had mild anemia and 3/10 (30%) had moderate anemia, and the median hemoglobin (Hb) level of all patients with UCD was 116 g/L (IQR: 97.55, 132; range: 78–147). A decreased mean corpuscular volume (MCV) was observed in 8/18 (44%) patients overall and 6/10 (60%) patients with anemia. The platelet count was increased in 11/22 (50%) patients, and the median count was 307 × 10^9^/L (IQR: 265.25, 447; range: 178–698). The median albumin level was normal (median: 42.7 g/L [IQR: 34.05, 45.12; range: 27.7–50]), whereas 7/21 (33%) patients had a decreased level. Additionally, 12/21 (57%) patients had an increased globulin level (median: 31.6 g/L [IQR: 25.35, 43.1; range: 20.6–84.9]). C-reactive protein (CRP) was measured in 15 patients and elevated in 5 (33%) (> 8 mg/L). An elevated IgG level was observed in 1/2 (50%) patients. Human immunodeficiency virus (HIV) antibody screening was negative in 19/19 patients. Epstein-Barr virus (EBV) IgG and Cytomegalovirus (CMV) IgG were detected in 6/6 patients and 4/6 patients, respectively, but the relevant lymph node staining was not performed on them. Patient 7 presented with positive anti-streptolysin O (ASO) and rheumatoid factor, and patient 22 had elevated levels of interleukin (IL)-8 and IL-12.

**Fig. 2:**
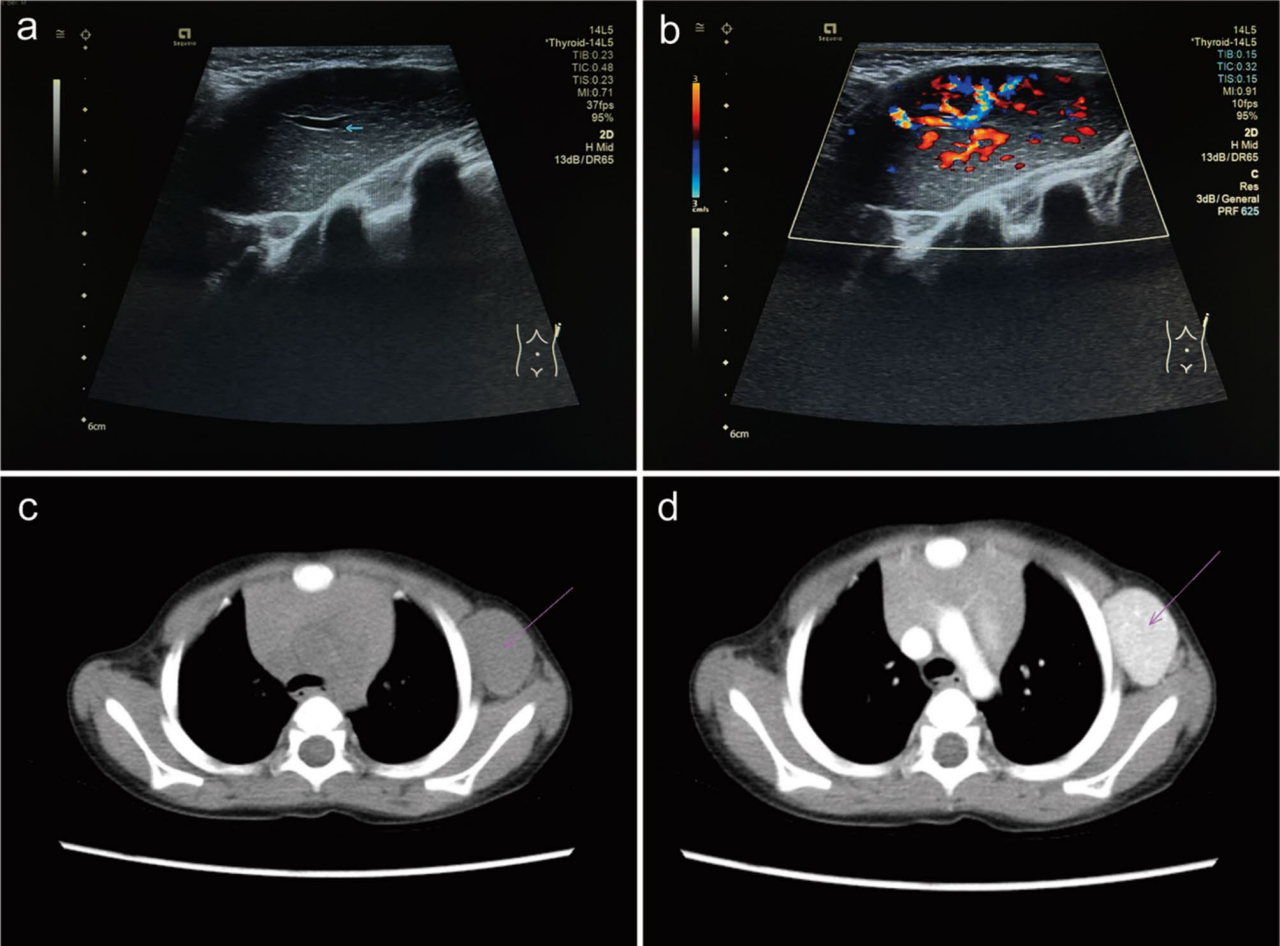
Representative imaging pictures of a left axillary mass in unicentric Castleman disease. **a** Ultrasonography image showed a homogeneous hypoechoic area with a regular shape and sharp border and a thick blood supply vessel passing through the mass (blue arrow); **b** color Doppler flow imaging detected rich blood flow signals in the mass; **c** general CT image showed a homogeneous mass with similar density to soft tissue (purple arrow), and the CT value was 55 Hu; **d** the mass was significantly and homogeneously enhanced after contrast injection, and the CT value was 160 Hu

Adjunct examinations were performed in 20/22 patients for diagnosis. Ultrasound was most commonly used, followed by computed tomography (CT) (16/20, 80%), magnetic resonance imaging (MRI) (6/20, 30%), and fine-needle aspiration cytology (FNAC) (3/20, 15%). As in adults, lesions imaged with ultrasound always demonstrated homogeneous hypoechoic nodules with rich blood flow signals. Mild to moderate homogeneous enhancement was a typical feature in CT and MRI. The mean CT value before and after enhancement in six patients was 47.6 (9.95) and 132.35 (19.63) HU, respectively. Representative images are displayed in Fig. 2.

All patients with UCD underwent surgical resection only. After a median follow-up of 4.08 years (IQR: 1.63, 6.08), all patients had survived and none had developed relapse or progression, 10/16 (63%) were in complete remission, and 6/16 (38%) had stable lymphadenopathy. Patient 10 was prone to colds and slight fevers, and patient 21 complained of fatigue. Patient 8 developed persistent numbness in her right hand after hospital discharge and had an intermittent headache for 5 days, two episodes of vomiting, and one episode of convulsions, which improved after symptomatic treatment.

### MCD

Both patients with MCD were male, presented the HV subtype, HIV negative, EBV IgG positive, and CMV IgG positive.

For patient 23, the age at diagnosis was 13 years and the diagnostic delay was 24 months. The primary lesion was a painless mass palpated in the neck. Upon diagnosis, no systemic symptoms were present. Multiple lymphadenopathies were detected in the IB and II regions in the bilateral neck, the V region in the right neck, and the right internal iliac region by positron emission tomography (PET)-CT. The Hb level (159 g/L), MCV (87.3 fL), platelet count (265 × 10^9^/L), CRP level (< 8 mg/L), albumin level (47.1 g/L), and globulin level (27.8 g/L) were normal, while the levels of IgG (15.43 g/L) and inflammatory cytokines including tumor necrosis factor (TNF)-α, interferon (IFN)-α, IFN-γ, and IL-8 were elevated.

For patient 24, the age at diagnosis was 15 years and the diagnostic delay was 5 days. The primary lymph node was located in the axilla and discovered during routine follow-up for connective tissue disorder (CTD). He had an EBV infection and presented EBER-positive. Fever and mild anemia were present at the time of diagnosis. PET-CT revealed involvement of lymph nodes, bones, bone marrow, spleen, and nasopharynx; hepatomegaly was also present. The CRP level was markedly high at 245.87 mg/L. Other laboratory findings were an Hb level of 107 g/L, MCV of 77.9 fL, platelet count of 313 × 10^9^/L, albumin level of 34.21 g/L, globulin level of 32.99 g/L, IL-6 level of 11.3 pg/mL, and IgG level of 18.77 g/L. Serum antinuclear antibody (ANA), ASO, and IL-8 were all increased.

Both patients with MCD underwent excisional biopsy and IHC examinations. Patient 23 was a consultation patient without access to subsequent treatment. Patient 24 received rituximab therapy once a week for 1 month postoperatively and then switched to thalidomide and dexamethasone. Some inflammatory indexes were abnormal during follow-up, including IL-6 (8.8 pg/mL), CRP (53.52 mg/L), and erythrocyte sedimentation rate (27 mm/h); however, no symptoms developed.

## Discussion

Our patients with UCD had a balanced sex ratio, consistent with previous reports [5, 6]. Although both patients with MCD were male, more research is needed to determine the sex distribution of MCD. CD can occur at any age, but the median age of patients with UCD is usually younger than that of patients with MCD [7, 8]. This is also supported by our results.

CD was originally described as HV and PC types with an intermediate mixed type. However, experts have proposed that the features present in the different subtypes occur along a spectrum of pathologies rather than fitting into three easily definable clusters [3]. UCD commonly presents as the HV subtype, whereas MCD commonly presents as the PC and mixed subtypes [3]. Nevertheless, both of our patients with MCD exhibited typical characteristics of the HV subtype, and up to 67% of patients with iMCD in a two-site pediatric cohort presented the HV subtype [5]. IHC has high sensitivity and is valuable for the differential diagnosis and histological profiling of lymphoproliferative diseases. Expressions of CD138 and VS38C can facilitate verifying the polyclonal nature of CD [9], discriminating it from neoplastic diseases. Especially, the proliferative plasmacytoid cells in HHV-8-MCD express monotypic but not monoclonal IgM-λ, due to up-regulation of recombination activation protein-mediated V(D)J recombination and Igλ expression in HHV-8-infected Igκ-naïve B lymphocytes [2]. Although the categorization can be helpful for the histopathological diagnosis of CD, it has an ambiguous clinical impact and cannot be utilized alone for clinical management.

UCD is generally considered to present a homogenous phenotype as asymptomatic isolated lymph nodes or symptoms associated with a mass effect. In our cohort, up to 45% of patients with UCD presented with systemic symptoms, similar to another pediatric cohort [5]. However, the proportion was significantly higher than that in adults at 18% [7]. One of our patients with UCD exhibited growth retardation, and a previous report also described a patient with UCD whose only symptom was short stature [10]. Growth inhibition may result from chronic inflammation mediated by cytokines, which is also attributed to delayed diagnosis. This might occasionally be the primary reason for hospitalization and could be a crucial presentation of CD in children.

Among our 22 patients with UCD, 9 had detectable symptoms including pallor, abdominal pain or discomfort, chest pain, fatigue, anorexia, and growth retardation, whereas the other 13 were asymptomatic. As shown in Table 3, the primary lymph nodes were located internally in all symptomatic patients but in only 2/13 (15%) of asymptomatic patients. Overall, peripherally located masses were easily detected, resulting in earlier access to medical care, shorter diagnostic delays, and less likely to be symptomatic. Conversely, our data suggested median diagnostic delays of 6 months (IQR: 1, 18) and 4.5 months (IQR: 0.85, 24) for the asymptomatic and symptomatic patients, respectively, but the difference was not statistically significant (*z* = 0.00, *P* = 1.00). The mean maximum dimension of single lesions in the two groups was 3.98 (0.96) and 5.23 (1.13) cm, respectively, and was larger in the symptomatic group (*t* = − 2.82, *P* = 0.01).

**Table 3:** Comparison of asymptomatic and symptomatic UCD patient

-	Asymptomatic UCD (*n* = 13)	Symptomatic UCD (*n* = 9)	*p*
Neck/submaxilla *n* (%)	9 (69.23%)	0 (0%)	-
Axilla *n* (%)	1 (7.69%)	0 (0%)	-
Mediastinum *n* (%)	1 (7.69%)	1 (11.11%)	-
Abdomen *n* (*n*%)	1 (7.69%)	8 (88.89%)	-
Groin *n* (%)	1 (7.69%)	0 (0%)	-
Diagnosis delay (month) Median (IQR)	6 (1, 18)	4.5 (0.85, 24)	*z* = 0.000
			*p* = 1.000
Maximum dimension of single lesion (cm) Mean ± SD (range)	3.98 ± 0.96 (2.95–6)	5.23 ± 1.13 (4–7.48)	*t* = − 2.822
			p = 0.0105

*UCD *unicentric Castleman disease, *IQR *interquartile range, *SD *standard deviation

MCD can be subdivided into HHV-8-MCD, iMCD, and POEMS-MCD. HHV-8-MCD is typically accompanied by immune impairment, most commonly HIV infection [4]. Both of our patients with MCD were HIV negative, but their HHV-8 infection status was unavailable. By comparison, adults with MCD have an 86.2% chance of being HHV-8 positive [7], whereas the proportion in children with MCD is always 0% [5, 6, 9]. This may be attributed to the varying prevalence of HIV and HHV-8 in different populations.

The diagnosis of MCD in patient 23 required differentiation from IgG4-related disease (IgG4RD) because IHC showed a high number of IgG4-positive cells (> 100/hpf) and elevation of serum IgG4. In addition, the lymphadenopathy and increased levels of serum inflammatory biomarkers observed in patient 23 were shared by iMCD and IgG4RD [11]. It is essential to distinguish between these conditions because of their different clinical courses and therapeutic strategies. The serum IgG4/IgG ratio is a more reliable discriminator than the absolute IgG4-positive cell count, which may be distinctive for IgG4RD when > 40% [11]. By contrast, a persistent rise in IgA, IgM, and CRP is an exclusive criterion for IgG4RD [11]. In our patient, the IgG4/IgG ratio was < 40% and the histopathologic findings were characteristic for the HV type; thus, the diagnosis of MCD was confirmed.

At diagnosis, patient 24 presented overlapping manifestations between MCD and CTD, including fever, anemia, and positive autoantibody (ANA 1:320 +). Autoimmune appearances can occur at any period in the course of MCD. In previous studies, 28% of patients with systemic lupus erythematosus [12] and almost all patients with rheumatoid arthritis [13] showed histopathological features comparable with those of MCD. Additionally, 30% of patients with iMCD showed autoantibody positivity or autoimmune hemolytic anemia [14]. These findings demonstrate that autoimmune abnormalities have a potential relationship with MCD, and clinicians should be aware of the possibility of the coexistence of autoimmune diseases and CD to improve the accuracy of diagnosis.

The underlying pathogenesis of CD has not been fully elucidated. Some data have demonstrated that UCD may be a neoplastic cloning process involving stromal cells, predominately follicular dendritic cells [2]. *PDGFRB* mutations located in CD45^−^ stromal cells were found in 17% of patients with UCD [15], and the allele frequency increased with the proliferation of stromal cells [16]. Uncontrolled HHV-8 infection and inflammatory storms involving cytokines such as IL-6 are crucial drivers of HHV-8-MCD and iMCD, respectively [2]. However, transcriptome sequencing showed that IL-6 expression was not significantly higher in iMCD-involved lymph nodes than in normal lymph nodes [17]. Notably, other cytokines including TNF-α, IFN-α, IFN-γ, and IL-8 were elevated in our patients, and their role in the disease course needs further verification. Among them, IFN-I has been proven to be involved in the pathogenesis of iMCD by increasing JAK/STAT-dependent activation of mTOR signaling [18]. Mutations in genes associated with autoinflammatory diseases may also be involved in the pathogenesis of iMCD [6]. The next step should be deeper investigation into the mechanism level.

There is no consensus regarding whether FNAC can serve as a definitive diagnostic modality. In the present study, 3/22 patients with UCD and 1/2 patients with MCD underwent FNAC, but none were conclusively diagnosed by FNAC alone. Distinguishing morphological features of CD include lymphocyte-dominant cell smears, an admixture of GC cells, PCs, and hyalinized capillary fragments with adhered lymphocytes [19]. Deeper insight into the subtle cytomorphological features of CD is essential to discriminate it from its mimics.

Regardless of the pathology, the gold standard treatment for UCD is surgical resection, which has a complete remission rate of 90%. For unresectable disease, chemotherapy, radiotherapy, or embolization should be considered [3]. For patients with HHV-8-MCD, a rituximab-based therapy regimen results in a 5-year OS rate of 90% [3]; however, there is no standard treatment regimen for iMCD. IL-6 inhibitors are the only first-line therapy for iMCD approved by the FDA [20], but up to two-thirds of patients have no response [21]. High IgG and fibrinogen levels imply a high response potential, whereas the opposite is true for high CRP and Hb levels [22]. A 17% reduction in CXCL13 by 8 days after siltuximab therapy is also a predictive indicator [23]. Therefore, serum index quantification can help guide and modify the treatment strategies. Likewise, rituximab was ineffective for patient 24 after just two doses, and a combination of thalidomide and dexamethasone kept him in stable condition. Recent clinical trials involving the thalidomide-cyclophosphamide-prednisone (TCP) regimen and the bortezomib-cyclophosphamide-dexamethasone (BCD) regimen have shown promising outcomes [24, 25]. The mTOR inhibitor sirolimus [26] and the JAK inhibitor ruxolitinib [27] have also benefited some patients. Such clinical trials in children are urgently needed to determine the efficacy and safety of these drugs. A clinical trial of sirolimus involving adults and children is ongoing (NCT03933904).

The prognosis of CD is more favorable in children than adults [5], and as expected, the progression-free survival rate in our study was 100%. In a CD cohort with the largest sample size to date, the 5-year OS rate was 95% and 74% for patients with UCD and MCD, respectively [8]. The PC type, hepatomegaly and/or splenomegaly, Hb of ≤ 80 g/L, and albumin of ≤ 30 g/L were recently confirmed as independent prognostic factors for OS, especially in patients with iMCD [8].

This study had some limitations. Because of its single-center, retrospective nature, bias in sample selection and data collection was inevitable. The panel of IHC was incomplete. Given the lack of samples, the calculation of mean values in the patients with MCD, and the comparison between two phenotypes were meaningless. However, our work effectively summarized the characteristics of children with CD to a certain degree, aiming to provide a reference for optimal management.

## Conclusion

We retrospectively analyzed a pediatric cohort with CD, in which HV-UCD was the most prevalent type. The children’s histopathological manifestations were relatively homogeneous. Diagnostic delay remains an unresolved issue. Surgical excision is the first-line treatment for UCD and has been proven quite beneficial. A standard therapeutic regimen for iMCD is needed, especially for patients who do not respond to IL-6 inhibitors. Although there was a high proportion of systemic symptoms among children with UCD, OS was still considerably high.

## Data Availability

The data that support the findings of this study are available from the corresponding author upon reasonable request.

## Abbreviations

ANA: Antinuclear antibody
ASO: Anti-streptolysin O
CD: Castleman disease
CMV: Cytomegalovirus
CRP: C-reactive protein
CTD: Connective tissue disorder
EBV: Epstein-Barr virus
FNAC: Fine-needle aspiration cytology
GC: Germinal center
Hb: Hemoglobin
HHV-8: Human herpes virus 8
HIV: Human immunodeficiency virus
HV: Hyaline vascular
IFN: Interferon
IgG4RD: IgG4-related disease
IHC: Immunohistochemical
IL: Interleukin
iMCD: Idiopathic multicentric Castleman disease
IQR: Interquartile range
MCD: Multicentric Castleman disease
MCV: Mean corpuscular volume
OS: Overall survival
PC: Plasma cell
POEMS: Polyneuropathy, organomegaly, endocrinopathy, monoclonal plasma cell disorder, skin changes
TNF: Tumor necrosis factor
UCD: Unicentric Castleman disease
